# Hand Warmer-Induced Hypoxia Accelerates Pest Control in Hermetic Storage

**DOI:** 10.3390/insects15100821

**Published:** 2024-10-20

**Authors:** Wenbo Li, John Stephen Yaninek, Dieudonne Baributsa

**Affiliations:** Department of Entomology, Purdue University, 901 W. State St., West Lafayette, IN 47907, USA; li2793@purdue.edu (W.L.); yaninek@purdue.edu (J.S.Y.)

**Keywords:** hypoxia treatment, cowpea bruchids, oxygen scavengers, grain storage

## Abstract

**Simple Summary:**

This study tested hand warmers as oxygen scavengers for controlling insect pests in hermetic storage by accelerating oxygen depletion. Using one, two, or four hand warmers, this study found that only four hand warmers effectively reduced oxygen below 1% and maintained it for up to 168 h. Insect mortality increased with more hand warmers and extended storage duration, achieving 100% mortality after 5 and 8 days for four and two hand warmers, respectively. Hand warmer treatments did not affect the moisture content or germination rates of cowpea seeds. Hand warmers effectively accelerate oxygen depletion, leading to high insect mortality and progeny suppression, without impacting grain quality.

**Abstract:**

Accelerating oxygen depletion during hermetic storage can minimize pest damage and preserve product quality. This study evaluated the effectiveness of hand warmers in accelerating hypoxia to control insect pests inside hermetic containers. We used one, two, or four hand warmers to deplete oxygen in a 4-gallon hermetic jar with 4 kg of cowpea and cowpea bruchids, alongside a non-hermetic control with cowpea bruchids and no hand warmers. Oxygen levels, insect mortality, egg counts, seed moisture content, and germination rates were monitored over 2, 5, or 8 days of storage. Only the four hand warmers treatment reduced oxygen levels below 1% within 12 h and maintained them for up to 168 h. The other treatments did not achieve this level. Insect mortality was higher with more hand warmers and extended storage duration, reaching 100% after 5 and 8 days with four and two hand warmers, respectively. Similarly, increased hand warmers and extended storage durations reduced egg counts and adult emergence. The treatments did not affect the moisture content or germination rates of the stored cowpea seeds. Hand warmers proved effective in accelerating hypoxia during hermetic storage, resulting in high insect mortality and reduced reproduction, without compromising grain quality.

## 1. Introduction

Post-harvest losses pose a significant challenge to farmers worldwide, impacting food security and income [1,2]. To mitigate these losses and improve food safety, hermetic storage technologies have been disseminated widely to prevent damage from insects and other living organisms [3,4,5,6]. Hermetic storage plays a crucial role in reducing grain losses by creating airtight conditions that preserve the quality of stored products [7]. Hermetic storage systems work by restricting air movement from outside to the inside of the container. Air trapped in intergranular space inside the hermetic container is depleted by insects and other living organisms [8]. When oxygen drops to 5% or below, insects cease feeding and reproducing, leading to no further damage.

Hermetic storage systems come in two main forms: flexible and rigid containers [6,9,10,11,12]. Rigid structures include silos, drums, and jerricans, while flexible storage containers include hermetic bags, Cocoons, and TranSafeliners. Although hermetic storage systems are highly effective, they face challenges that can affect their performance. Residual intergranular airspace in hermetic storage containers may provide insects with enough oxygen to potentially survive longer. When the infestation levels are low, oxygen depletion takes longer, increasing the risk of further infestation. Additionally, in areas with moderate temperatures, the rate of oxygen consumption inside hermetic bags slows down, prolonging insect survival [13,14]. This delay in oxygen depletion may result in potential grain damage before insect pests succumb to oxygen deprivation.

Several studies have investigated methods for removing residual oxygen from hermetic containers, exploring various technologies and strategies. For example, oxygen scavengers have been shown to effectively reduce residual oxygen levels in grain storage bags [15]. In addition, hand warmers have proven effective as oxygen scavengers, accelerating oxygen depletion in hermetic storage systems [16]. Air-activated hand warmers work through a chemical reaction between iron powder and oxygen, resulting in iron oxide (rust) [17]. This process leads to a depletion of oxygen in the surrounding environment. Furthermore, germinating seeds have been tested and found to absorb the residual oxygen through respiration [18,19]. In metal silos, a common practice has involved lighting a candle inside the rigid container to consume the remaining oxygen [20,21]. However, this practice cannot be carried out in flexible or rigid plastic containers to avoid damage to these structures. Nitrogen and carbon dioxide have been used in laboratory settings to displace residual oxygen in the intergranular space [22,23].

Despite these promising findings, there is a notable gap in the research. Specifically, no study has thoroughly examined the potential of using hand warmers not only to accelerate the removal of residual oxygen but also insect mortality. Rapid oxygen reduction could potentially enhance insect mortality rates and suppress progeny development. In this study, we evaluated the effect of accelerated hypoxic conditions induced by hand warmers on pest control by assessing (i) oxygen concentration, (ii) adult insect mortality, and (iii) adult insect oviposition and progeny development. We also evaluated the effect of hand warmers on grain quality by assessing the moisture content and germination rate of stored cowpea seeds.

We hypothesized that the accelerated hypoxic conditions created by hand warmers could effectively speed up insect mortality, inhibit reproduction, and preserve the quality of stored grain. This research addresses several gaps in enhancing efficacy and expanding the use of hermetic storage. Specifically, we explore whether hypoxia induced by hand warmers can (i) serve as a one-time treatment for infested grains or (ii) accelerate hypoxia to eliminate insect infestations during storage. The results of this study could benefit farmers in developed countries, where tolerance for insects is minimal, who are interested in using hermetic containers to store specialty crops (grains, seeds, or other products).

## 2. Materials and Methods

This research was conducted from spring 2024 to summer 2024 in the Post-harvest Innovation for Crop Storage Laboratory at Purdue University (West Lafayette, IN, USA).

### 2.1. Insect Rearing

Cowpea bruchid (*Callosobruchus maculatus*) colonies were reared in a Conviron insect growth chamber (Model CMP4030; CONVIRON, Winnipeg, MB, Canada) for 30 days before the experiment. A vacuum aspirator was used to remove adult cowpea bruchids from pre-established colonies. The remaining infested cowpea seeds were then transferred to a 2 L jar and placed in a growth chamber maintained at 25 ± 1 °C and 40 ± 5% RH for 48 h. Newly emerged insects from these seeds were separated with a size 6 sieve to create a cohort of 2-day-old adults. Ensuring the uniform age of adult cowpea bruchids was essential to reduce age-related differences in insect mortality measurements. Female and male cowpea bruchids were identified by the shape, size, and pattern of the abdomen and elytra. Females have an ovoid shape, featuring a larger, black-marked plate at the end of the abdomen and elytra longer than 1 mm. In contrast, males have a rounded shape with a smaller, stripe-less plate and elytra shorter than 1 mm.

### 2.2. Experimental Set-Up

HotHands^®^ hand warmers with 10 h capacity from Kobayashi Healthcare International, Inc. (Dalton, GA, USA) were obtained online from Amazon.com, Inc. Airtight 4-gallon (15,200 mL) glass jars (Daitouge, China) were purchased online from Amazon.com, Inc. https://www.amazon.com/dp/B09S3973D9?ref_=cm_sw_r_cp_ud_dp_09WX386SJ64BAE1JEQPR (accessed on 3 February 2023). Untreated seeds of black-eyed cowpea (*Vigna unguiculata*) were obtained from MBS SEED, Inc. (Denton, TX, USA).

**Figure 1:**
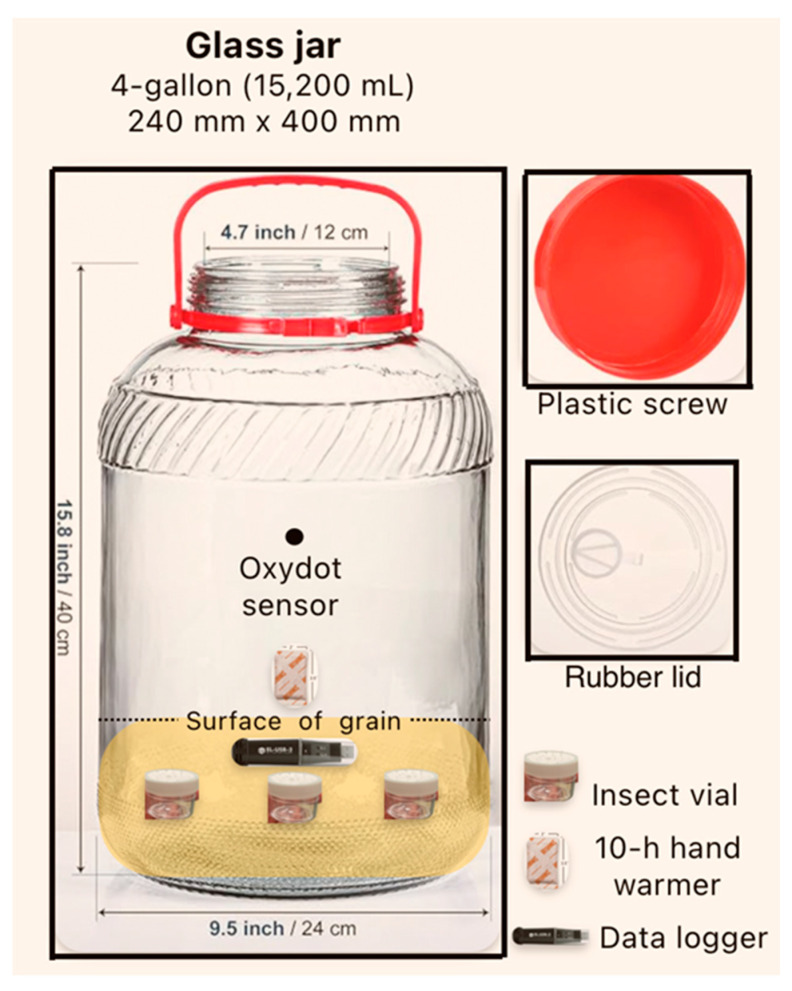
Schematic representation of an experimental 4-gallon glass jar. Source: Adapted using images from Amazon and Lascar Electronics.

The treatments consisted of 1, 2, or 4 hand warmers placed in 4-gallon glass jars, each containing 4 kg of cowpea seeds. All hand warmers were placed on top of the grain (Figure 1). Because we lacked larger hermetic containers, the 4-gallon jars were only filled to approximately 25% of their capacity to exhibit significant differences among hand warmer treatments (Figure 1). The purpose of adding cowpeas was to leave enough air space for the hand warmer treatments to deplete oxygen yet provide a suitable environment for the insects. Three 30 mL wide-mouth plastic vials with mesh lids were placed in the upper column of the grain in each replicate or 4-gallon jar (Figure 1). Each vial contained 10 insects (5 females and 5 males) and 10 cowpea seeds as a substrate for oviposition. Hence, each replicate had 30 insects (15 females and 15 males). The control with no hand warmers comprised three replicates of non-hermetic 4-gallon jars, each containing 4 kg of cowpeas and 9 vials (with 10 insects each). The 9 vials were the pseudo-replicates of the treatment jars (3 treatments × 3 replicates). Three sets of treatment jars (3 replicates per treatment) and one control jar were opened after 2, 5, and 8 days of storage. At each opening, 3 vials from the control were assigned to each treatment. There were 3 treatments with 3 replicates each, 3 storage durations, and 3 control jars, for a total of 30 jars.

### 2.3. Data Collection

Oxygen concentration, temperature, and relative humidity: The OxySense^®^ 525OI Oxygen Analyzer (Industrial Physics, Devens, MA, USA) was used to monitor the oxygen concentration in the hermetic jars. Data were not collected on non-hermetic jars because no changes were expected in oxygen concentration. An Oxydot sensor was affixed to the wall inside each hermetic jar (Figure 1). A fiber optic oxygen reader connected to the oxygen analyzer quantified alterations in intensity and fluorescence properties of Oxydot sensors, which measured the real-time oxygen concentration inside the containers. The oxygen concentration in the hermetic jars was monitored at 1 h intervals over the first 12 h and every 24 h thereafter for 8 days. One data logger (EL-USB-2, Lascar Electronics Inc., Erie, PA, USA) was set to record the temperature and relative humidity in 30 min intervals in each jar (Figure 1). Meanwhile, one data logger was placed in the same room outside the jars to monitor ambient room temperature and relative humidity conditions.

Insect mortality: A selection of hermetic jars for each treatment with three replicates and one control were opened after 2, 5, and 8 days of storage to collect data (destructive sampling). For each opening, a vial containing insects was taken out of each container for evaluation while the hand warmers were discarded. Insects in each vial were poured onto a white paper to determine the survival of adult cowpea bruchids. Adult cowpea bruchids showing any sign of movement were considered alive. Inactive adults gently probed repeatedly with a camel hairbrush were placed back in a growth chamber at 25 ± 1 °C and 40 ± 5% RH for an additional 2 h to confirm their death [18]. All adults were discarded after assessing their mortality. The cowpea seeds with eggs laid on the surface were kept for egg counting and incubation.

Egg counts and progeny development: Cowpea seeds from each vial were inspected for egg counts. Eggs on the seed surface were counted using a hand lens with a 5X magnification under directed lighting. In addition, eggs laid on the interior surface of each vial were also recorded. Eggs laid per female were determined by dividing the total number of eggs recorded in a vial by 5 (the total number of females per vial). After counting, cowpea grains with eggs from each treatment were then placed back in their respective vials with mesh lids and kept in a Caron insect growth chamber (Model 6025-1, 115 VAC, Caron Growth Chambers, Marietta, OH, USA) set to 25 ± 1 °C and 40 ± 5% relative humidity. Following a 35-day incubation period, the number of emerging adult insects was recorded daily for up to 60 days in both the treatments and the controls.

Seed moisture content and germination rates: Cowpea seed moisture content was determined by the oven-dry method on a wet basis [24], where the grain weight was measured before and after drying. Three 15 g seed samples were taken from each jar and dried in a tin cup at 103 ± 1 °C for 72 h. The difference in the dry weights represents the moisture content. The cowpea germination test followed the International Rules for Seed Testing protocol [25]. Four 25-seed samples were taken from each jar, giving 100 seeds. Each 25-seed sample was distributed uniformly in a Petri dish lined with damp filter paper, which was kept moist to promote germination. The number of successfully germinated seeds per Petri dish was logged daily for seven days to gauge the overall quality of the seeds.

### 2.4. Data Analysis

All statistical analyses in this study were performed using Prism-GraphPad version 10.0.3. Tukey’s multiple comparison test was performed to compare the means of oxygen concentration, insect mortality, egg counts, progeny development, temperature, relative humidity, and moisture content. Means were separated with a confidence level of 95%. A multiple linear regression model was used to study the relationship between oxygen concentration, hand warmers, and time. A correlation was performed to examine the relationship among oxygen concentration, insect mortality, egg counts, number of hand warmers, and storage duration in 4-gallon hermetic jars filled with 4 kg cowpea seeds. All graphs were made using Microsoft Excel 2016.

## 3. Results

### 3.1. Oxygen Concentration

**Figure 2:**
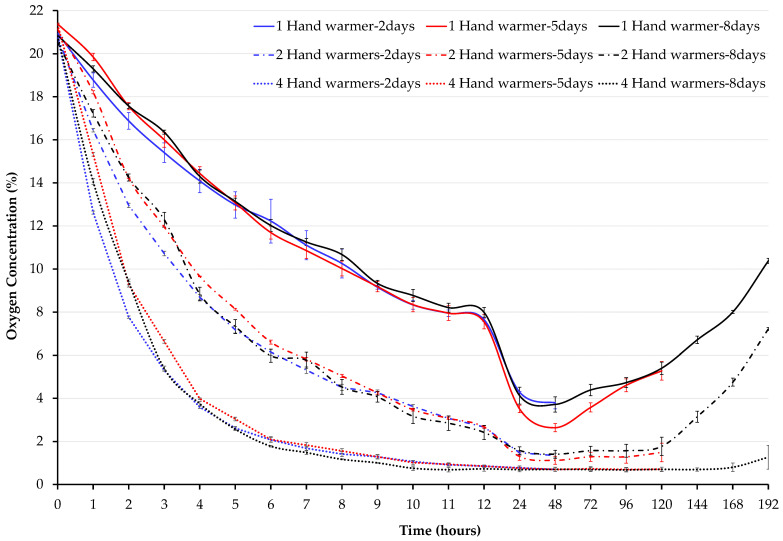
Oxygen concentration inside hermetic jars stored for 2, 5, and 8 days (*n* = 3). A 4-gallon jar containing 4 kg of cowpea and adult bruchids in vials was used for each treatment (1, 2, or 4 10 h hand warmers) in hermetic or non-hermetic (control) storage. Data were not collected on non-hermetic jars because no changes were expected from the ambient oxygen concentration (20.9%). Each error bar represents the standard error of the mean (SEM).

The oxygen concentration for 1, 2, or 4 hand warmers varied in hermetic jars stored for 2, 5, and 8 days (Figure 2). There were significant differences among hand warmer treatments during the first 48 h for jars stored for 2, 5, and 8 days (F = 81.52, *p* < 0.0001), and between 72 and 120 h for jars opened after 5 and 8 days of storage (F = 6.33, *p* = 0.0001) (Table 1).

During the first 48 h, oxygen concentration was affected by the number of hand warmers (F = 387.3, *p* < 0.0001) and storage duration (F = 10,612, *p* < 0.0001). All hand warmer treatments reduced the oxygen concentration to below 5% within 24 h (Figure 2). Regardless of the storage duration, the one, two, and four hand warmer treatments reached below the 5% threshold within the first 24, 8, and 4 h, respectively. For the treatments opened after 5 and 8 days of storage, the one and two hand warmers treatments maintained an oxygen concentration below 5% for up to 96 h and 168 h, respectively, while the four hand warmers treatment remained below the 5% threshold over 8 days (Figure 2). Only the four hand warmers treatment reached less than 1% (Table 1). This 1% level was reached within the first 11 h and maintained until 168 h.

The effect of hand warmers and time on oxygen concentration was modeled using a multiple linear regression estimated for the period from 0 to 48 h, where all three hand warmer treatments were functioning within their capacity. These data reflect values from all hermetic jar treatments where a significant interaction was found between treatment and time (F = 394.7, *p* < 0.001) and an adjusted R squared of 90%. We found that oxygen concentration was reduced with increased hand warmers, as noted by the negative slope estimates (Table 2). There was a negative 4.36 and 7.51 percentage point drop in oxygen concentration as the number of hand warmers increased from 1 to 2 and 4, respectively.

### 3.2. Insect Mortality

Insect mortality of adult bruchids stored in hermetic jars was significantly affected by both the number of hand warmer treatments (F = 753.4, *p* < 0.05) and storage duration (F = 149.4, *p* < 0.05). Table 3 indicates that insect mortality increased with the number of hand warmers and the storage duration. The two and four hand warmers treatments achieved complete adult mortality after 8 and 5 days of storage, respectively (Table 3).

### 3.3. Egg Counts and Progeny Development

**Figure 3:**
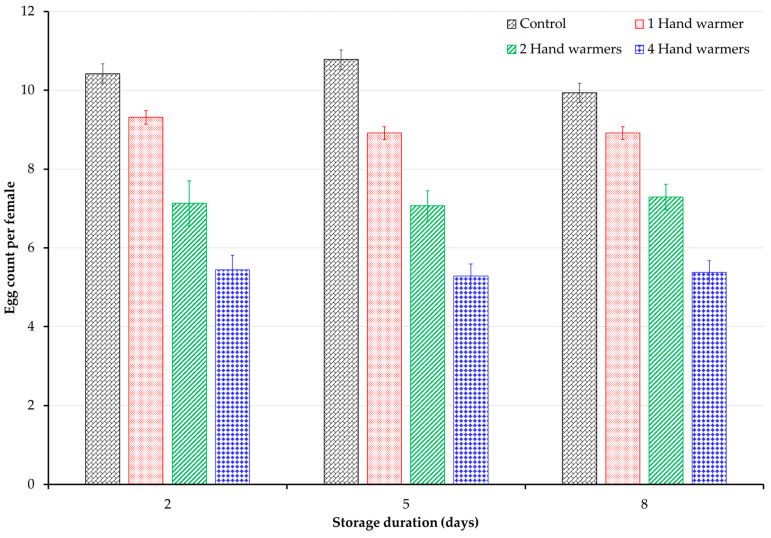
Average number of eggs laid per female *C. maculatus* on cowpea in vials kept in hermetic and non-hermetic control jars after 2, 5, and 8 days of storage. A 4-gallon jar containing 4 kg of cowpea and adult bruchids in vials was used for each treatment (1, 2, or 4 10 h hand warmers) in hermetic or non-hermetic (control) storage.

The number of eggs laid per female cowpea bruchid was significantly affected by the number of hand warmer treatments (F = 109.7, *p* < 0.001), but not by the storage duration (F = 0.44, *p* = 0.595). Treatments and control exhibited similar patterns for egg counts across the three storage durations (Figure 3). When comparing within each storage duration, egg counts generally decreased with increased hand warmers (Figure 3). However, some similarities were observed between treatments, as no significant difference was noted between the two and four hand warmers treatments after 2 days of storage and between the one hand warmer treatment and the control after 8 days of storage.

The adult emergence rates of cowpea bruchid were significantly affected by both the number of hand warmer treatments (F = 1314, *p* < 0.001) and storage duration (F = 75.49, *p* < 0.001 (Table 3). The control consistently exhibited higher adult emergence rates than all other treatments across all storage durations. In general, increasing the number of hand warmers and extending the storage duration significantly reduced the adult emergence rates. No adults emerged in jars containing two hand warmers stored for 8 days, and those containing four hand warmers stored for 5 and 8 days (Table 3).

### 3.4. Relative Humidity and Temperature

While the relative humidity in the room fluctuated over time, the average RH inside the jars containing hand warmer treatments and the control, stored for 2, 5, and 8 days, remained relatively constant (Table 4). The average relative humidity was 55.44 ± 0.71%, 53.07 ± 0.29%, 55.54 ± 0.87%, and 55.08 ± 0.66% for the control, and one, two, and four hand warmers, respectively. There was no interaction between treatments and storage duration (F = 4.63, *p* < 0.0026). Relative humidity varied among treatments (F = 13.43, *p* = 0.0005) and storage duration (F = 107.9, *p* < 0.001). The average temperature was 21.78 ± 0.51%, 21.96 ± 0.45%, 22.05 ± 0.42%, and 21.88 ± 0.46% for the control, and one, two, and four hand warmers, respectively (Table 4). There was an interaction between treatments and storage duration (F = 10.82, *p* < 0.0001). Temperature varied among treatments (F = 8.00, *p* = 0.0037) and storage duration (F = 1114, *p* < 0.0001).

### 3.5. Seed Moisture Content and Germination Rate

No changes were observed in cowpea seeds’ moisture content and germination rates before and after the experiment. Moisture content of the stored cowpea seeds was not affected by hand warmer treatments (F = 1.15, *p* = 0.39) nor storage duration (F = 1.64, *p* = 0.22), and there was no interaction between treatments and storage duration (F = 0.99, *p* = 0.47). Initial cowpea grain moisture content was 8.89 ± 0.07, 8.85 ± 0.07, and 8.86 ± 0.06% after 2, 5, and 8 days of storage, respectively. It fluctuated between 8.84 ± 0.13 and 8.90 ± 0.06%, 8.66 ± 0.01 and 8.92 ± 0.08%, and 8.77 ± 0.04 and 8.88 ± 0.07% after 2, 5, and 8 days of storage, respectively. Similarly, germination rates of cowpea seeds were not affected by hand warmer treatments (F = 1.56, *p* = 0.26) or storage duration (F = 0.82, *p* = 0.45). Also, there was no interaction between the treatments and storage duration (F = 0.71, *p* = 0.68), initial germination rates were 100% for the cowpea stored for 2, 5, and 8 days. It remained at 100% for the control and 99.67 ± 0.33 after 8 days of storage, but varied between 99.00 ± 0.58 and 99.67 ± 0.33, and 99.33 ± 0.67 and 100.00 ± 0.00% after 2 and 5 days of storage, respectively.

## 4. Discussion

This study is the first to explore the use of commercial hand warmers in hermetic storage to accelerate oxygen depletion and speed up insect mortality. In our study, the four hand warmers treatment reduced oxygen levels to 1% or lower within 12 h and maintained these levels for up to 168 h (7 days). In comparison, two hand warmers achieved oxygen concentrations between 1% and 2% after 24 h and sustained this range for 120 h (5 days). Our results show that increasing the number of hand warmers accelerates oxygen reduction in hermetically sealed jars and significantly improves the overall efficiency of oxygen depletion. Based on regression analysis, doubling the number of hand warmers from 2 to 4 led to a 72.3% increase in the reduction in oxygen concentration. Hand warmer treatments were found to be faster than modified atmosphere methods, mostly driven by insect respiration, which take 5 to 8 days to reach 1% [9,23]. In contrast to controlled atmospheres, which rapidly reduce oxygen levels to below 1% by displacing air with gases like nitrogen or carbon dioxide [22,26,27,28], the use of four hand warmers achieved similar results in just 12 h. The increase in oxygen concentrations after 5 days could likely be due to a minor leakage in the hermetic jars over time, as observed in previous studies [28,29].

Low oxygen levels induced by four hand warmers resulted in complete adult insect mortality within 5 days, while two hand warmers required slightly longer (8 days) to achieve the same outcome. Previous studies have shown that exposure to hypoxic conditions below 1% generally leads to high insect mortality in a short period [18,22,28,30]. Extended exposure to oxygen levels below 5% also effectively controls cowpea bruchids [8,27]. Prolonged exposure to low oxygen conditions is lethal not only to cowpea bruchids but also to other stored product pests [30,31]. Research indicates that oxygen levels below 1% can halt insect activity and lead to 90% mortality within 4 days [22,27]. Faster oxygen depletion and longer storage duration contribute to higher mortality rates in cowpea bruchids. Our findings are reinforced by strong correlations: a negative correlation between oxygen concentration and insect mortality (r = −95%), and positive correlations between both storage duration (r = 64%) and the number of hand warmers (r = 70%) with insect mortality.

In addition to increased mortality, cowpea bruchid oviposition was suppressed by the accelerated low oxygen levels induced by hand warmers. The number of eggs laid by a cowpea bruchid significantly decreased with an increasing number of hand warmers. This is evidenced by strong correlations between both the number of hand warmers (−82%) and oxygen concentration (83%) with egg counts. There was minimal variation in egg counts across different storage durations within each treatment. This suggests that female cowpea bruchids likely dumped most of their eggs in the first initial hours or days due to rapidly changing hypoxic conditions. Stressful environments, such as hypoxia, negatively impact reproduction by suppressing oviposition [28,32]. Previous studies on hypoxia in cowpea bruchids indicated that female reproductive output remains impaired even after oxygen levels return to normal conditions [26,28].

Similar to the impact on egg counts, progeny development was also suppressed by the accelerated hypoxic conditions created by hand warmers. Treatments with more hand warmers and longer storage duration reduced adult insect emergence, as indicated by the strong negative correlation between storage duration and adult emergence (r = −0.74). Previous studies have reported similar results when low oxygen levels were achieved through controlled atmosphere methods such as gas flushing and seed germination [19,31]. Correlation analysis further underscores the importance of egg count and oxygen concentration on progeny development. Oxygen concentration and adult emergence had a strong positive correlation (r = 0.99). Additionally, the significant correlation between egg count and post-treatment adult emergence (r = 0.78) further supports these findings. Overall, these results confirm that doubling hand warmers not only enhanced oxygen reduction but also accelerated hypoxic conditions, effectively suppressing insect oviposition and progeny development.

Although statistically significant differences were observed in the means of relative humidity and temperatures among treatments and storage duration, these changes were numerically minimal. The variations within each treatment across storage durations are likely attributed to the data being collected on different dates. The primary purpose of monitoring environmental conditions was to determine if the heat from the hand warmers would significantly raise the temperature inside the hermetic containers. The findings of this study show that the heat generated did not cause any noticeable temperature increase, even with more hand warmers. The fluctuations in relative humidity had minimal impact on seed moisture content and germination rates, as no significant changes were observed before or after storage nor among treatments. Consequently, the data on moisture content and germination rates support our hypothesis that accelerated hypoxic conditions created by hand warmers had no effects on stored grain quality.

Hand warmers effectively accelerated oxygen depletion in hermetic containers, leading to faster pest control and preservation of grain quality. A higher number of hand warmers rapidly achieved very low oxygen levels (below 1%). However, storage duration appears more critical in suppressing progeny development than the number of hand warmers used. For instance, two hand warmers with longer storage durations produced similar results in adult mortality and emergence as four hand warmers with shorter storage durations. Given the cost implications, it is worth exploring the effectiveness of using fewer hand warmers over longer storage durations to achieve insect mortality and suppress progeny development. Hand warmers can be a viable option for protecting grain or seed stored in small containers, such as the 4-gallon jars used in this experiment. In addition, testing hand warmers’ effectiveness in larger containers is essential to determine the appropriate number needed for various sizes of commercially available airtight storage (e.g., 50 versus 100 kg hermetic bags). Unlike gases such as nitrogen or carbon dioxide, hand warmers are easy to use as they do not require specialized equipment. However, their cost-effectiveness should be compared to that of these gas treatments.

## 5. Conclusions

This study demonstrates that hand warmers used as oxygen scavengers effectively accelerate hypoxic conditions within hermetic storage, leading to rapid insect mortality and suppressed progeny development. Importantly, the results showed no adverse effects on cowpea seeds’ moisture content or germination rates. Data on insect mortality and progeny development suggest that two hand warmers used over longer storage durations achieved similar outcomes to four hand warmers with shorter durations. While further research is needed, hand warmers show potential for insect pest control in long-term hermetic storage or as a treatment for infested grain. Using enough hand warmers to create lethal hypoxic conditions can effectively stop the infestation. Though these results are promising, the number of insects used (30 per replicate, or 90 per treatment) falls slightly below the phytosanitary threshold, which typically recommends at least 120 insects to ensure treatment effectiveness under various conditions and to meet international standards. Future studies should increase the insect count to at least 120 per treatment, as larger sample sizes would provide more robust data and better validate the effectiveness of hand warmers as oxygen scavengers in hermetic environments. Additionally, studies are required to assess the applicability of these findings in field conditions.

## Figures and Tables

**Table 1:** Average oxygen concentration (O_2_%) for the first 48 h inside hermetic jars stored for 2, 5, and 8 days. A 4-gallon jar containing 4 kg of cowpea and adult bruchids in vials was used for each treatment (1, 2, or 4 10 h hand warmers) in hermetic or non-hermetic (control) storage. Data were not collected on non-hermetic jars because no changes were expected from the ambient oxygen concentration (20.9%).

		Oxygen Concentration (%, Mean ± SEM) *						
					Time			
Storage Duration	Treatment	0 h	3 h	6 h	9 h	12 h	24 h	48 h
2 days	1 hand warmer	20.95 ± 0.13 b	15.4 ± 0.46 a	12.23 ± 1.02 a	9.16 ± 0.09 a	7.64 ± 0.07 a	4.29 ± 0.04 a	3.79 ± 0.28 a
	2 hand warmers	20.78 ± 0.13 b	10.72 ± 0.1 c	6.14 ± 0.15 b	4.26 ± 0.13 b	2.67 ± 0.05 b	1.47 ± 0.11 b	1.34 ± 0.11 c
	4 hand warmers	20.83 ± 0.06 b	5.3 ± 0.04 d	2.07 ± 0.11 c	1.29 ± 0.08 c	0.85 ± 0.05 c	0.78 ± 0.09 c	0.69 ± 0.1 e
5 days	1 hand warmer	21.39 ± 0.12 a	16.0 ± 0.02 a	11.69 ± 0.01 a	9.18 ± 0.14 a	7.55 ± 0.11 a	3.51 ± 0.41 a	2.64 ± 0.71 b
	2 hand warmers	21.13 ± 0.05 a	11.95 ± 0.01 b	6.6 ± 0.09 b	4.26 ± 0.07 b	2.67 ± 0.05 b	1.3 ± 0.17 b	1.12 ± 0.19 d
	4 hand warmers	21.34 ± 0.05 a	6.64 ± 0.08 d	2.09 ± 0.13 c	1.29 ± 0.1 c	0.85 ± 0.05 c	0.76 ± 0.1 c	0.69 ± 0.1 e
8 days	1 hand warmer	20.85 ± 0.16 b	16.36 ± 0.09 a	12.02 ± 0.28 a	9.33 ± 0.14 a	7.99 ± 0.23 a	4.13 ± 0.39 a	3.72 ± 0.35 a
	2 hand warmers	20.55 ± 0.03 c	12.29 ± 0.35 b	5.97 ± 0.3 b	4.07 ± 0.24 b	2.42 ± 0.33 b	1.58 ± 0.17 b	1.4 ± 0.19 c
	4 hand warmers	20.81 ± 0.13 b	5.39 ± 0.11 d	1.78 ± 0.04 c	1.00 ± 0.01 c	0.72 ± 0.11 c	0.69 ± 0.1 c	0.7 ± 0.1 e

* All data are means ± standard error of means (SEM). Under the same time, means within the same column followed by the same letter are not significantly different (*p* < 0.05).

**Table 2:** Multiple regression of oxygen concentration (%) as a function of the number of hand warmer units (1, 2, or 4) and time (log_hour) with estimated marginal slopes, standard error, t, and *p*-values. A 4-gallon jar containing 4 kg of cowpea and adult bruchids in vials was used for each treatment (1, 2, or 4 10 h hand warmers) in hermetic or non-hermetic (control) storage.

Variable	Estimated Marginal Slopes	Standard Error	t Value	*p* Value
Intercept	17.92	0.37	49.05	<0.001
1 Hand warmer	0.00	.	.	.
2 Hand warmers	−4.36	0.43	10.21	<0.001
4 Hand warmers	−7.51	0.43	17.59	<0.001
Time (log_hour)	−8.49	0.29	29.53	<0.001

**Table 3:** Average insect mortality and adult emergence of *C. maculatus* in cowpea stored in hermetic and non-hermetic control jars after 2, 5, and 8 days. A 4-gallon jar containing 4 kg of cowpea and adult bruchids in vials was used for each treatment (1, 2, or 4 10 h hand warmers) in hermetic or non-hermetic (control) storage.

		Storage Duration		
Variable	Treatment	2 Days	5 Days	8 Days
Insect mortality (%)	Control	0.00 ± 0.00 cA *	0.00 ± 0.00 dA	3.33 ± 1.67 cA
	1 Hand warmer	24.44 ± 2.94 bC	45.56 ± 2.42 cB	54.44 ± 1.76 bA
	2 Hand warmers	35.56 ± 4.12 bC	72.22 ± 4.01 bB	95.56 ± 2.42 aA
	4 Hand warmers	66.67 ± 3.73 aB	96.67 ± 2.36 aA	100.00 ± 0.00 aA
Adult insect emergence (%)	Control	89.27 ± 3.68 aA	89.24 ± 1.43 aA	86.67 ± 1.32 aA
	1 Hand warmer	63.62 ± 10.17 bA	34.67 ± 6.02 bB	26.56 ± 3.68 bB
	2 Hand warmers	54.00 ± 2.35 bA	4.00 ± 4.74 cB	0.00 ± 0.00 cB
	4 Hand warmers	35.67 ± 8.86 cA	0.00 ± 0.00 cB	0.00 ± 0.00 cB

* All data are means ± standard error of means (SEM). Within the same variable, means among treatments (lower-case letter) and storage durations (upper-case letter) followed by the same letter are not significantly different (*p* < 0.05).

**Table 4:** Average relative humidity (%) and temperature (°C) in hermetic and non-hermetic control jars after 2, 5, and 8 days of storage. A 4-gallon jar containing 4 kg of cowpea and adult bruchids in vials was used for each treatment (1, 2, or 4 10 h hand warmers) in hermetic or non-hermetic (control) storage.

		Storage Duration		
Variable	Treatment	2 Days	5 Days	8 Days
	Ambient room	56.20 ± 0.07 aA *	56.09 ± 0.03 aA	54.02 ± 0.02 aB
Humidity (%)	Control	53.44 ± 0.32 bB	55.16 ± 0.17 aA	51.58 ± 0.14 bC
	1 Hand warmer	53.40 ± 0.07 bA	53.33 ± 0.04 bAB	52.48 ± 0.06 bB
	2 Hand warmers	56.19 ± 0.07 aA	56.62 ± 0.05 aA	53.82 ± 0.02 aB
	4 Hand warmers	55.54 ± 0.06 aA	55.92 ± 0.03 aA	53.77 ± 0.03 aB
	Ambient room	22.53 ± 0.05 aA	22.00 ± 0.03 aB	20.80 ± 0.03 cdC
Temperature (°C)	Control	22.04 ± 0.05 bA	21.45 ± 0.04 bB	20.65 ± 0.03 dC
	1 Hand warmer	22.80 ± 0.09 aA	21.83 ± 0.06 abB	21.25 ± 0.06 abC
	2 Hand warmers	22.87 ± 0.12 aA	21.83 ± 0.07 abB	21.46 ± 0.06 aC
	4 Hand warmers	22.71 ± 0.10 aA	21.80 ± 0.05 abB	21.12 ± 0.04 abcC

* All data are means ± standard error of means (SEM). Within the same variable, means among treatments (lower-case letter) and storage durations (upper-case letter) followed by the same letter are not significantly different (*p* < 0.05).

## Data Availability

Raw data are not publicly available but may be obtained upon request.
